# Who Put the Super in Superhero? Transformation and Heroism as a Function of Evolution

**DOI:** 10.3389/fpsyg.2018.02514

**Published:** 2019-01-11

**Authors:** Susan L. Ross

**Affiliations:** Department of Health Science and Recreation, San Jose State University, San Jose, CA, United States

**Keywords:** spiritual growth, evolution-cosmology, supernatural, psychological transformation, transpersonal anthropology, alchemy, hero's journey, Aurobindo

## Abstract

Transformation and heroism are reciprocally related. Transformation produces an individual that others may call hero; one who inspires, guides, and protects something precious—an ordinary extraordinary person, master of the self. Heroes exhibit the further reaches of human development by transforming into entirely new, resplendent individuals that demonstrate valuable capacities whiles still being mortal. Because transformation is the means through which heroes are made, a more thorough understanding of the forces affecting transformation may advance collective understanding of the demands upon the individual. Founded on the scholarship of seminal authors of depth psychology, East Indian spirituality, anthropology, physics, mythology, Hermetic science, and other disciplines, this paper argues that a hero develops through both natural and supernatural processes, which can eventually produce a transhuman or superhero—depending on one's perspective, one who is natural (bound by nature) and supernatural (beyond nature). If this psychospiritual proposition has merit, advancement from ordinary to hero and from hero to super hero—each resulting from trans-form-ation—constitutes a subtle yet fundamental change in form, a microevolution of a subsect of individuals. The progressive framework begins with a description of patterns of evolution salient to heroism and transformation; an exploration of four key “laws” that govern the realm of matter, which support and delimit the budding hero as an earth-bound human; and an examination of four “supernatural” abilities the initiate must cultivate and enact in order to transform. The heart of the paper is a detailed guide identifying when during the process of transformation, the initiate can expect to receive the support of natural processes, when to be vigilant for beyond nature or Divine intervention, and when to intervene with “supernatural” activities. The framework culminates with an elucidation of how the ordinary yet heroic individual becomes a superhero or transhuman as outlined by spiritual teacher Sri Aurobindo, through three transformations of biopsychosocial maturation and spiritual realization.

Transformation and heroism are reciprocally related. Transformation produces an individual that others may call hero; one who inspires, guides, and protects something precious—an ordinary extraordinary person, master of herself^1^. The mere thought of one's hero lifts the spirit, enlivens the heart, illumines the mind, and rouses the body to move courageously into previously unknown territories. Heroes inspire ordinary people through the trials of transformation because they demonstrate it is possible to emerge as triumphant; to inhabit valuable capacities while still being mortal.

Scholars and untrained people alike have gained considerable understanding about the making of heroes (van Gennep, 1909/1960; Eliade, 1958/2005; Campbell, 1968; Turner, 1969; Franco and Zimbardo, 2006; Allison et al., 2017; Ross, 2017), and yet our collective knowledge of “how heroes are created…remains a critical area of future research” (Jayawickreme and di Stefano, 2012, p. 174). Because transformation is the means through which heroes are made, a more thorough understanding of the forces affecting transformation, may advance collective understanding of the demands upon the individual. Specifically, it is not known if the process of transforming from ordinary person to hero follows the laws of nature that govern human development, causing heroic aspirations to be within everyone's grasp. Or, if heroism is a progression beyond nature (supernatural), a transcendental process that only the fortunate can apprehend. Or, a combination of the two possibilities. Furthermore, the progression to become a hero constitutes human advancement and as such, it is plausible that the hero will, one day, endeavor once again to transcend all that she knows. If she does, will she remain a hero in the eyes of those who love her or advance and become a superhero and if so, how will we distinguish her? One postulation suggests that super heroism is not simply a fantasy but a certainty; that the average person is “a transitional being….not final….the step from man to superman is the next approaching achievement in the earth's evolution” (Sri Aurobindo, 1972, p. 7).

This article contributes to the literature gap on how heroes are created by exploring the following proposition: heroism and human transformation require evolutionary processes that are both *natural* (limited to the laws of nature) and *supernatural* (above nature, pertaining to the Divine). Together, these produce a hero: a human who is literally part natural and part supernatural. This analysis draws upon discourse in depth psychology (Jung, 1988), anthropology (Turner, 1969), mythology (Campbell, 1968), Hindu spirituality (Sri Aurobindo, 1972), physics (Prigogine, 1997), and the Hermetic Sciences (Eberly, 2004). This inquest contains three main sections: (1) a discussion of select concepts and terms pertaining to the qualities and movement of evolution, natural, and supernatural phenomena as related to transformation; (2) an explanation of the timing, in terms of when transformation processes are governed by nature, when the hero must grow beyond nature and act with super-natural capacities, and when Divine forces intervene; and finally, (3) a conceptualization of evolution from novice, to hero, to superman or superwoman, as achieved through three substantial transformations of biopsychosocial maturation and spiritual realization.

## Evolution as it pertains to transformation

Because initiates become heroes through transformation, and transformation results in a refined, more evolved creature, it is useful to review some fundamental concepts related to the purpose and movements of evolution. Each point will be elaborated in a later discussion. First, what is the initiate evolving *from* and *toward*? Philosopher and teacher G. I. Gurdjieff explains, “In order to know one cosmos [reality], it is necessary to know the two adjoin cosmoses” (Ouspensky, 1949/2001, p. 206). Ancient and modern cosmologies and depth psychology uphold an ontology that humans have access to and can even exist (albeit for most, unconsciously) within three worlds: the world of matter within which we live; a lower or inner world that can be qualified as the shadow, underworld, darkness, or subconscious; and an upper, celestial, heavenly world of a higher or increased consciousness (Ouspensky, 1949/2001; Sri Aurobindo, 1972; Jung, 1988).

Second, what aspect of the initiate is evolving or transforming? Seminal authors across diverse disciplines agree that the transforming feature is primarily the initiate's *consciousness* (Ouspensky, 1949/2001; Newman, 1978; Jung, 1988; Wilber, 1996; Prigogine, 1997), which is defined here as a force or power comprised of two binary capacities or compositions: discrimination and unity (Sri Aurobindo, 1972). These two capacities cause a dynamic tension—an individual possessing consciousness will have capacities to discriminate between that which is self and that which is not self. Prior to consciousness, the entity projects self onto objects, and there is no distinction between self and object. The projection, due to a lack of consciousness, is the seed of all opposites, including the notion of good and evil. Despite these abilities of division, a person with consciousness will also be able to sense through division and experience the unity inherent in all, and will be able to unify perceptions and self (Sri Aurobindo, 1972; Jung, 1988).

Third, what is the *context within which the initiate is developing*? East Indian spiritual teacher and author Sri Aurobindo offers an understanding of the evolution of the universe that also describes the evolution of individual consciousness as an inevitable and natural process. According to Aurobindo, all of existence is an eternal and infinite “Unmanifested Supreme” (Sri Aurobindo, 1972, p. 24), the Divine or God identified by many religious and secular names across time. In this Unmanifested state, the original Divine (a substance-force) initiated evolution that began with involution: a descent of itself as consciousness into matter (Ouspensky, 1949/2001, p. 134). When consciousness emerged out of unconscious matter for the first time, the world split into an endless assortment of pairs of opposites. Once consciousness arrived into the lowest point, into all of the darkest, most unconscious, inert, and motionless of substances on earth, it began a great, slow ascent outward and upward, liberating the “latent indwelling spirit” existing within matter (Sri Aurobindo, 1972, p. 19). Because consciousness is moved by its impulse to emerge (Wautischer, 2008, p. 476) from that within which it dwells, everything in existence will eventually blossom because the Divine “aspires to become its real self by transcending its apparent self” (Sri Aurobindo, 1972, p. 53). From these perspectives, the impulse to transcend, go beyond the known self, is impelled by an evolutionary compulsion.

This explanation of evolution supports Jung's proposition that “All initiations we know by history or by experience are the external manifestation of a *natural* inner process which is *always* happening” (italics added; Jung, 1988, p. 236). Like nature, Jung explains, a person's unconscious mental faculties are in “a process of continuous transformation” (p. 236) of which nothing is realized because the unconscious material is not made conscious:

No crops are brought home by nature; only the consciousness of man knows about crops. He gathers the apples under the trees for they simply disintegrate if left to themselves. And that is true of our unconscious mental process: it revolves within itself. It builds up and it pulls down; it integrates and disintegrates—and then integrates again. (p. 236)

Here Jung describes rhythms of nature and uses the word *integration* to describe an organic process where life coagulates to produce fruit—different from integration as a psychospiritual process of incorporating experience and energy into the psyche and body. Jung (1988) identifies the continuous ebb and flow of creation as unconscious, “a building up and pulling down, integration and disintegration without end” (p. 236); the birth-death-rebirth succession that “needs our conscious interference to bring it to a goal….Otherwise, it is like the eternal change of the seasons in nature…from which nothing comes unless a human consciousness interferes and realizes the result” (p. 237); by the harvesting of crops. Jung's analogy demonstrates how the initiate can actively engage in her own evolution by paying attention to her inner world and by contemplating and incorporating insights—as they arise and emerge from the unconscious—into her ontology, her experience of what is real or true.

In a context of growth, as caused by a descent of consciousness into matter and an emergence of consciousness out of matter, the hero-initiate is innately compelled to become conscious, to allow consciousness to emerge from within. Evolution causes the initiate to grow, learn, and encounter life-changing experiences—albeit unconsciously at first—and to eventually become a master herself, a hero. The drive to learn and grow, to become a healthier, happier, more triumphant self is, according to these perspectives, the impulse that drives and enables the initiate to be grounded in and limited to the middle or natural world, able to sink inward to the lower world or shadow, and also to rise beyond (or above) the self into the upper world or Divine. In order to set the four “super-natural” abilities necessary for this process of hero-transformation, I first describe four rules of nature that support and delimit the initiate.

### Rules of Nature Affecting Hero-Transformation

Humans view activity as supernatural (above nature) when the action does not comply with the “laws of nature,” but those laws are part of a particular perspective. From the outlook of the lower world, our own (middle) world is above or beyond what is natural, and from the viewpoint of the world above human life—the supernatural realm—ours appears lower or unconscious. With this distinction in mind, this section reviews four “rules” of nature (as viewed from the realm of matter) that influence and constrain the hero. These “rules” in particular serve as the source of the hero's earth-bound humanness. The hero is restricted in that, in the end, the hero remains human and yet the task is to apprehend the undifferentiated Divine; meaning humans are “rendered frustrate by the very organs through which the apprehension must be accomplished (Campbell, 1968, p. 258).

The first relevant “rule” of nature, as shown by the Nobel Prize winning theory of Dissipative Structures, states that *chaos is critical to the transformation of a system*—that “dynamical instability provides only those conditions necessary to generate evolutionary patterns of nature” (Prigogine, 1997, p. 128). Just as elements of the universe transform through chaos, so too must the initiate enter “dynamical instability” or personal challenges in order to transform. Interestingly, this theory shows how matter that is close to equilibrium (a period of peaceful balance) is “‘blind,ș but far from equilibrium…[in the midst of chaos or liminal state] it begins to ‘see”ș (p. 67). This knowledge translates into social science (Prigogine, 1997), and in a heroism context, indicates that the hero-transformation process must include instability and disequilibrium.

The second “rule” of nature relevant to transformation is that *the initiate is continuously affected by unforeseen circumstances arising from the unconscious self*, best described by Gurdjieff as “the law of accident” (Ouspensky, 1949/2001, p. 199). In this esoteric teaching, humans who are unconscious (i.e., not awakened in consciousness) are subject to moment-by-moment encounters with incidents and activities that arise organically, due to pure chance. For example, an individual might have a plan to go to the grocery store, but later visit a relative when unforeseen circumstances arise—her car needed more gasoline, the store was out of the item she needed, and it began to rain heavily—all of which caused a delay in her plans. Gurdjieff states that an unconscious individual maintains an illusion of having the ability set a goal and achieve tasks and aspirations, when in actuality, she is at the mercy of myriads of circumstances, none of which is within her control. This ontology is similar to what Jung referred to as the “building up and pulling down” (1988, p. 236) referred to earlier, meaning the unconscious ebb and flow of creation. Inclusive of the “law of accident” are the two phases of nature, variously described as “ascending and descending….contraction and expansion” (Burckhardt, 1997, p. 44), integration and disintegration” (Jung, 1988, p. 1402), and “dissolution and coagulation” (Burckhardt, 1997, p. 123). As a part of nature, humans participate in these continuous movements of the “undulating sea of the unconscious” (Burckhardt, 1997, p. 153). The potential is for the hero-initiate to transform to the degree that she supersedes the law of accident, which allows her to live deliberatively, co-creating her moment-to-moment living.

A third “rule” pervades all levels of existence and is integral to the heroic journey: *the primordial cycle of life, death, and rebirth*. The anthropologist Eliade (1958/2005) explains that the desire to transform is “far more than the obscure desire of every human soul to renew itself periodically” rather, the desire to transform is embedded in universal passages of life-death-rebirth, just “as the cosmos is renewed” (p. 135). This cycle is exhibited in the “two phases of nature,” which are “dissolution and coagulation,” where new life dissolves until it's death and coagulates to reform new life. This universal cycle sustains the movement of physical and psychological realms that encumber shifting from darkness into light and back again into darkness.

The ubiquitous Daoist symbol Yin Yang depicts this natural rhythm using symbols of the Chinese classic *Book of Changes*, known as the I-Ching. As Jung explains, in this philosophy,

Yang eats the Yin, and from the Yang, Yin is reborn; it bursts forth again, and then Yin envelops the Yang, and so on. *That is the course of nature*. ….spirit eats the flesh and then the flesh eats the spirit. (emphasis added; Jung, 1988, p. 67)

The alchemical symbol of the snake eating its tail, *ourobóros*, reflects the cyclical nature of the endless drawing together of creation into a substance and then dissolving back unto itself (van der Sluijs and Peratt, 2009). Sometimes depicted as a circle or an infinity shape, it is also a symbol of individuation and exemplifies wholeness as exhibited in the Model of a Complete Transformation (Ross, 2017).

The fourth and most concrete “rule” is that *the hero has physical (mental and emotional) limitations and as such, she depends upon the earth and others, to survive*. Humans have naturally occurring personal weaknesses, which they may or may not overcome. It has been suggested that that the body, mind, and psyche must be made ready for transformation—that a person cannot withstand transformation unless she advances her holistic health through disciplined action (Ouspensky, 1949/2001; Sri Aurobindo, 1972; Jung, 1988). In the end, our frailties—the aspects of self of which we are less capable—define our humanness. Although “nature seeks and demands a gradual attainment of perfection, and a gradual approximation to the highest standard of purity and excellence” (Henry, 1893/2012, p. 16), the imperfection existing in the realm of matter—and in us all as human beings—remains critically grounding. Our limitations can indeed be our commonality, what connects us one to another as humans. Accepting weaknesses helps the hero-initiate not only to cultivate compassion and humility, but also to develop supernatural capacities that transform limitations into strengths. By knowing and accepting her humanness, the initiate is poised to develop skills to go above or beyond nature, into the super-natural.

### Supernatural Abilities Necessary to Hero-Transformation

Because humans can act with unconsciousness or consciousness, routes toward transformation are optional—an initiate must draw on supernatural abilities in order to complete her transformation into a hero. When an individual survives unconsciously, she is at the mercy of and pace of natural evolution (Jung, 1988; Prigogine, 1997)—but when an initiate chooses to open into vulnerability towards personal growth (caused by a lack of control) she will eventually transform, and be liberated into active co-creation of the self. According to Sri Aurobindo (1972), two aspects of human beings are “supernatural,” existing beyond the realm of nature in the upper world: consciousness and ego (p. 57). This is so because, to our knowledge, living entities other than humans do not demonstrate the capacity for self-reflexivity or an ego. Evolution (and therefore transformation) must engage the growth of both, because consciousness is a structure of the universe and ego is a structure of the human. Initiates hoping to transform can accelerate the process by taking action that is supernatural (Jung, 1988). In my investigation, I found four human abilities that engage consciousness and ego, and are therefore supernatural and support transformation.

The first supernatural ability occurs when the initiate has *moment(s) of self-awareness*. The impulse to learn about one's self, heal wounds, and mature psychologically comes from the emerging consciousness within, that desires “to be wholly aware of its objects…and the first [object] is the self” (Sri Aurobindo, 1963/1990, p. 119). Because the ego begins as an unconscious entity, it “resides” in the lower world of the unconscious. Jung explains that the premature ego “has no feeling of its own existence” (Jung, 1988, p. 935), and thus the goal of ego integration is for the ego to be established and secure that it exists. In order for the ego to know that it lives, it must be separate from nature, go against nature; “a certain amount of immorality and disobedience is absolutely necessary” (Jung, 1988, p. 936). In order for the ego to develop and mature, for example, one must go beyond the self into another “object” (i.e., step into someone else's shoes), to gain perspective and in so doing, become more aware or conscious of one's self. Any time a person acts with consciousness as in this example, as opposed to unconsciously led action, she goes outside of natural conditions.

The second supernatural ability builds upon the first, and occurs when a person *knowingly chooses to participate in a frightening activity in order to gain a growth-producing experience*. Jung describes this ability as “doing the things of which one is afraid, which only a human being will do” (Jung, 1988, p. 938), and offers the following example in nature.

Animals refrain from doing things they are afraid of, while man quite naturally asserts the Divine quality of his ego by doing just the things he is afraid of. That is so very much against nature that it is the strongest evidence of the autonomous existence of the ego and of the freedom of the human will. (p. 938)

Jung suggests that fearless action toward personal growth is the best evidence of an autonomous ego. In order for the ego to develop and consciousness rise, the initiate must face her shadow (rejected or shunned aspects of the self), again and again, in order to witness and eventually accept all aspects of her unconscious as part of herself. The process of seeing rejected and wounded aspects of the self is psychologically painful and causes suffering. Interestingly, Jung characterizes suffering as “detachment from nature, from unconsciousness, from the animal and the plant” (Jung, 1988, p. 938). Humans instinctively fear suffering and tend to avoid it at all costs. Yet, moving toward this fear and into suffering—the hurts and pains that are inevitable by virtue of being human—is integral to development and paramount to transformation. It is so integral, in fact, that consciousness cannot come into existence without this type of emotional suffering (Sri Aurobindo, 1972; Jung, 1988; Burckhardt, 1997). So, choosing to step into or accept fear in order to grow is a supernatural ability that supports transformation.

The third supernatural capacity necessary to hero-transformation is *creative activity* (Ouspensky, 1949/2001; Jung, 1988; Campbell, 1991; Prigogine, 1997), meaning any thought or action that is inspired from within and results in idea(s), word(s), or object(s) that did not previously exist *in the initiate's personal “world*.” This latter point is significant: she might create an idea that has been imagined by someone else previously, but the key to supernatural activity is that fact that *she created the thought within herself*; that the thought was authentically her own and not that the idea is unique in the world. Physicist Prigogine (1997) states that “human creativity and innovation can be understood as the *amplification of the laws of nature* already present in physics or chemistry” (emphasis added; p. 71), confirming cosmological ontologies which state that creativity is beyond or stretches the bounds of what is natural (Ouspensky, 1949/2001; Sri Aurobindo, 1963/1990; Jung, 1988). Jung says, “the human ego cannot live without creativeness; it proves its existence by inventing something, by doing something on its own out of the ordinary” (1988, p. 938). The danger of this type of growth, Jung warns, is that with every creative act there is inflation of the ego. When the initiate engages in creative activity, she strengthens her ability to engage in the supernatural activity of co-creation—bringing something into existence—allowing consciousness to emerge and extend beyond her into a new creation. When the initiate becomes a superhero and galvanizes this capacity, she will bring into existence ideas and objects that change entire communities, countries, or even the world.

Finally, with this fourth supernatural ability, the initiate must *produce outcomes deemed by self or others to be miraculous*. A *miracle* is defined as an event that is not understood based on natural or scientific laws; miracles are the realm of the Divine and are often considered the province of saints and angels. If, however, miraculous events are viewed from the perspective of the three different worlds discussed above, other considerations become possible. Gurdjieff and others explain that “[t]he broadening of consciousness does not proceed in one direction only, that is, in the direction of the higher cosmoses; in going above, at the same time it goes below” (Ouspensky, 1949/2001, p. 207). In other words, although there is an impulse upward when humans evolve, that growth moves in both directions. Thus, the hero-turned-superhero lives in three worlds and can operate under the differing and even opposing laws of the above and below. When she takes an action that is congruent to the upper or lower realm, it would appear to others in the middle realm as being contrary to natural laws. Perhaps this is why the ancient Peruvian Incan cosmology states that the aim of human development is to become a tree: it drinks light from the sun (upper world), is sustained by the minerals and water of the earth (lower world) and lives on earth (personal conversation, Juan Nuñez del Prado, May 2010).

## Natural, Divine, and Supernatural Forces in Hero-Transformation: A Matter of Timing

In order to achieve a transformation from hero to superhero, it is helpful to know when the time is ripe to use and develop one's supernatural capacities. In order to talk about that timing, I first make explicit the other “players” that contribute to the process. A careful analysis of literature revealed that the process of hero-transformation requires three “participants” or forces: nature or the natural world, the Divine, and humans with their capacity for supernatural actions (as discussed above). Each of these forces is primary to particular phases of transformation; understanding how these forces interact with the process of transformation does much to illuminate how initiates become heroes and when they play an active role.

**Figure 1:**
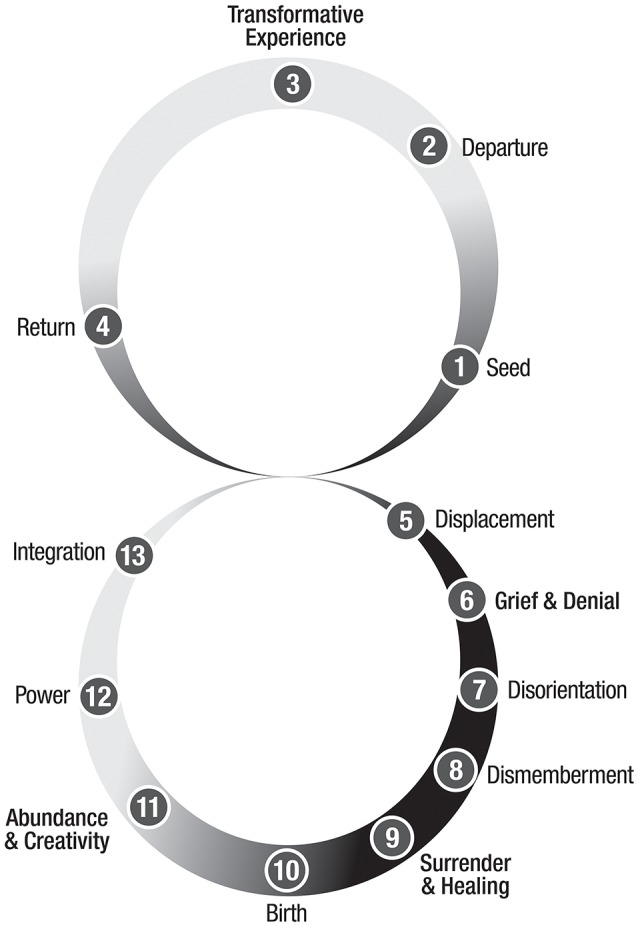
Phases and movement of a complete transformation. Copyright Susan Ross, 2018.

In my previous work, I identified a process of transformation that unfolds in the shape of a “Figure 8” or upright infinity symbol, with the upper loop (or cycle) as the transformative journey containing a transformative peak or trauma, and the lower loop as an integrative pathway that includes dismemberment (and death of some aspect of the self), healing, and rebirth (Ross, 2017) (Figure 1). This theory of transformation consists of 13 phases, 4 in the upper loop that develop and refine one's masculine, and 9 in the lower loop that develop and refine one's feminine (Ross, 2017).

The primary purpose of transformation is to transmute the ego, mind, or body and thereby re-create the structure of the self (Ross, 2019). During the upper transformative loop, the initiate expands beyond her body—beyond form—through a *trans-form*-ative experience where she contacts her soul (in the upper world), returns into the body (middle world of matter), and then journeys into her darkness or unconscious (lower world). The lower world experience leads to dissolution of the outdated structure of the self, and integration of both internal opposites and the expanded consciousness received during the transformative peak or trauma experience. Table 1 lists the 13 phases of a complete transformation and the primary forces for each.

**Table 1:** Phases and forces of transformation.

**No**.	**Title**	**Description**	**Forces**
**UPPER TRANSFORMATIVE LOOP—DEVELOPS AND REFINES THE MASCULINE**			
1	Seed	The seed that rests within the unconscious—and contains the latent yet complete version of the new self—awakens.	Nature, Divine
2	Departure	The initiate leaves home literally or figuratively to embark on a journey to find the self.	Nature
3	Transformative Catalyst	The initiate expands or shatters into and intermingles with the soul.	Nature, Divine
4	Return	The initiate moves homeward toward ordinary routine life.	Nature
**LOWER INTEGRATIVE LOOP—DEVELOPS AND REFINES THE FEMININE**			
5	Displacement	Upon return, the initiate is relieved and pleased to be in the familiar.	Nature
6	Denial and Grief	The initiate vacillates between denial and heartache, either that the experience is over (peak) or that it happened (trauma).	Nature
7	Disorientation	The initiate becomes disillusioned about his or her identity and about much if not most of his or her reality (i.e., purpose, relationships, career).	Supernatural
8	Dismemberment	Life circumstances cause the individual to lose a sense of control and to descend into a deeply distressing period of darkness, loss, and coming to terms with inner truths.	Divine, Supernatural
9	Surrender and Healing	The initiate releases ingrained dysfunctional patterns, and life circumstances help heal emotional pain and suffering.	Supernatural
10	Birth	Conceived during the peak or trauma, the new self is born.	Divine
11	Abundance and Creativity	The initiate receives support, resources, and relationships to engage in creative endeavors which he or she loves.	Nature
12	Power	The initiate achieves self-realization and the complete blossoming of his or her gifts, work, and relationships.	Nature
13	Integration	The initiate enjoys an inner state of homeostasis, balance, harmony, and bliss that permeate his or her outer life circumstances.	Nature

### Timing for Natural Processes

Nine of the 13 phases of transformation are governed by patterns heavily reliant upon the unconscious or latent consciousness of nature. Therefore, these phases transpire with little to no conscious effort: the initiate can enjoy the momentum and influences of nature as it incrementally progresses her. Phases 1–6 serve an important function of preparing the initiate's consciousness and ego for a transformation of the ego (or the mind or body, as discussed below). Phases 11–13 solidify the new ego through experiences that refine personally unique capacities and the knowledge that she has cultivated (and integrated) throughout the transformation process.

The upper transformative loop (phases 1–4) can be a cyclical pathway of expanding personal development, healing, and learning that incrementally progresses skills, consciousness, self-awareness, and the power to create and influence—all-the-while, cultivating the ego to maturity or integration (Loevinger, 1976). Humans depend on the ego in order to remain coherent in character, in separateness, and in “a sense that you exist in yourself” (Sri Aurobindo, 2002, p. 107), such that premature annihilation would mean “melting away…dissolving in a common mass of physical vibrations” (Sri Aurobindo, 2002, p. 107). For this reason, the first undertaking toward transformation is to develop the ego, which involves the challenging task of finding “a real personality in all… [the] forces movements, desires, [and] vibrations” that do not originate from within (Sri Aurobindo, 2002, p. 111).

The challenge with the ego is that as soon as it attains some semblance of strength, the individual can be misled by a false “sense of their importance and their ability [such] that they no longer even think at all of getting rid of their ego” (Sri Aurobindo, 2002, p. 105). Jung warns that an important part of the problem is an untamed or undisciplined mind that jumps from moment to moment, consumed by whatever is demanding attention. This type of constant movement “settles down and bounces off in the next moment” (Jung, 1988, p. 1391), and when all the activity commences, there is no change in consciousness. Jung cautions that the initiate will “never reap their crops…they plant their fields and then leave them behind before they are ready for harvest” (p. 1391), also dodging the responsibility of tending the crops and land while the crops grow. If nature is left to its usual progression, it “would be a movement without sequence [chaos],” lacking a “light [awareness and understanding] to its own mystery” (Sri Aurobindo, 1972, p. 19), or in other words, “If left to itself the [natural] process would come to nothing” (Jung, 1988, p. 237).

If the ego and consciousness are not developed enough to endure Dismemberment in phase 8, the transformation cycle is aborted and the initiate will again begin moving through phases 1–6. To maintain steady progress, Jung emphasizes, the initiate must pay attention to bodily “sensation[s]” (1988, p. 1392), the ways in which the body communicates emotions and psychological insights or awareness about others and one's self. He warns the initiate not to “overleap the body” (p. 1392), and instead to care for and tend to the body as she would a loved one. Incremental healing and growth reconfigure the ego, mind, and body for a complete transformation with the hope that consciousness emerges before “the ego becomes not only useless but harmful” (Sri Aurobindo, 2002, p. 108). Fortunately, Jung (1988) notes that if the initiate misses the opportunity for development and does not apprehend the transformative creation (i.e., self-awareness or in the case of the metaphor, harvesting the crop), the “same revelations [of fruiting crops or insight arise again at another time] without any issue” (p. 236). In the future, she can be confident that, due to nature, there will be countless small (yet important) opportunities to break through the bounds of unconsciousness and grow, as well as a number of life-changing opportunities where she can access wisdom and transform.

Nature also plays a dominant role during the final phases of transformation. During Abundance and Creativity (phase 11), Power (phase 12), and Integration (phase 13), the initiate reaps the rewards of having accomplished changes internally and in one's daily life. Again, the movement of nature carries the initiate forward: she needs simply to live life to the fullest, and natural circumstances will guide her through the final three phases to integration and the realization of a complete transformation.

### Timing for Divine Intervention

Four of the 13 phases of transformation (1, 3, 8, and 10) require Divine input as a catalyst. The evolutionary spark of consciousness emerging out of body, mind, or soul inspires insight, awakened sensations, awe, beauty, truth, or other experiences of illumination. Only the Divine has the ability to break “through the barrier of createdness” (natural laws) so that “humanness merges into divinity” (Mechthild, 1963/1993, p. 55). Hermetic science (also called alchemy) was the ancestor of modern chemistry and influenced the development of Western understanding of the world through figures such as Ralph Waldo Emerson (Emerson and Versluis, 1997), Carl Jung (1988), and Stokes (2010). According to this science, “Ancient and spiritual epitaphs propose that transformation requires divine aid…[where the goal] is obtained not by the might of man, but by the grace of god” (Olsen, 1992, p. 74). Aligned with Jung's teachings 1988, the same author forewarns that humans “can do nothing but sow, plant, and water: God must give the increase” (Olsen, 1992, p. 36).

Phase 1: Divine aid is what originates the process of transformation in the initial Seed phase, where the dormant, entirely whole, but not yet manifested self is triggered to awaken. In the Seed phase, the initiate is the “substance that is to be transformed” (Jung, 1988, p. 1407) and is “made ready…by the creator” (Henry, 1893/2012, p. 250). The Seed phase is, for most if not all individuals, entirely unconscious.

Phase 3: During Transformative Catalyst (phase 3), the initiate has a transformative experience, peak, or crisis. From a psychospiritual perspective, the life-changing event is a catalyst because “a descent of the Divine Nature can alone divinize the human receptacle” (Sri Aurobindo, 1972, p. 71). The transformative experience places the initiate in direct contact with a previously unavailable world:

Human consciousness can only attain dominion over the undulating sea of the unconscious with the awakening of a creative power within it, which derives from a higher sphere than that of ego–consciousness. This higher sphere…is pure undivided light…inaccessible to psychological observation. (Burckhardt, 1997, p. 153)

When this exchange with the “higher sphere” or Divine occurs, the body, mind, and spirit are prepared, but the initiate is not yet transformed.

Phase 10: Birth (phase 10) also necessitates Divine intervention. Of the thirteen phases, Birth offers the first visible, tangible sign that deep within the initiate, evolution has been unfolding. In the context of personal transformation, this phase constitutes the transition between involution (the infiltration of the Divine into the initiate's body) and evolution, “a gradual emergence of higher powers of consciousness leading to an even greater manifestation of the Divine consciousness-Force” (Sri Aurobindo, 1963/1990, p. 137) In other words, the new self is made manifest. When the soul replaces the ego and is seated in the body, the initiate has a direct connection to the ultimate source—“You carry the Divine within you, you have only to enter within yourself and you will find Him” (Sri Aurobindo, 2002, p. 94). When this happens, as Jung says, “Now we have reached something” (Jung, 1988, p. 237).

### Timing for Superhuman Action

Three phases of transformation *require* humans to take supernatural (or conscious) action. Throughout Disorientation (phase 7), Dismemberment (phase 8), and Surrender and Healing (phase 9), the initiate must partake in supernatural efforts to achieve the culminating and rigorous task of dissolving the ego. In particular, the initiate must regularly apply the supernatural skills of *conscious awareness* during honest self-appraisal and of *facing fears*, in order to apprehend the shadow and cause the ego to see itself.

Phase 7: Disorientation engages the initiate's experiences of facing fears as the ego becomes destabilized through challenges that cause confusion and instability, mainly about self-identity. Many situations arise for self-appraisal that cause the initiate to either enact desired healthier habits that are aligned with the emerging identity or revert backwards into unhealthy choices made into habit by the outgoing, dissolving identity. The initiate is overwhelmed and ill equipped to consummate what she truly desires, because she has not yet birthed the emerging identity that is capable of completing the goal. If the initiate's consciousness and ego develop through this demanding period, there is a juncture or bifurcation (Prigogine, 1997) and she advances, naturally, into the next phase.

Phase 8: Heroes must be conceived, and this “conception must comply with the same laws that Nature prescribes…dissolve and coagulate” (Eberly, 2004, p. 16). Readiness to enter into Dismemberment (phase 8) or dissolution, depends entirely on the initiate's previous conscious development, or ego strength. If the initiate requires further ego and consciousness maturity, she will unknowingly end this transformative process and soon begin a new transformation cycle.

The structures [i.e., the ego in this case] will not self-destruct simply because the soul has seen the light [during phase 3]. This is due to the fact that these structures and issues have mostly unconscious underpinnings. Unconscious elements of the psyche are not impacted by conscious experience directly, except maybe in exposing them to consciousness in some occasions. These structures are impacted only by awareness of them and complete understanding of their content. (Almaas, 2004, p. 194)

Thus, although revealed in phase 3, the ego structures will not dissolve (as required for transformation) until they are witnessed by the self during integrative processes of phases 7-9; the ego must become aware of itself fully.

If the initiate enters Dismemberment, she must enact the supernatural capacities of facing fears and witnessing consciously, as the self and life that she has known die. These processes ensure that “all that is impure and unsuitable…[is] purged off, and rejected like dross” (Henry, 1893/2012, p. 64). There is no way to avoid a nadir of suffering, because its purpose is to dissolve a human structure (ego, mind, and/or body) where “the body becomes the vessel for an incarnation of which we become one” (Jung, 1988, p. 200), meaning an integrated whole, a conscious being. Although no one wishes to endure such experiences, “entry into this obscurity, this void, this silence is only the passage to a greater existence” (Sri Aurobindo, 1963/1990, p. 122).

Phase 9: During Surrender and Healing (phase 9), the initiate discovers that when all “resistance is utterly gone, then the manifestation of the new symbol [the new ego, mind, or body structure] can take place” (Jung, 1988, p. 976). This phase also asks the initiate to face fears through surrender to circumstances that offer the potential for growth, and to engage in conscious awareness of the inner and outer worlds of synchronicities, feelings, sensations, and insights, but is considerably less painful than the previous phase. From the perspective of Hermetic Science, the initiate “*solve et coagula*, he dissolves the imperfect coagulations of the soul, reduces the latter to its *materia* [its most irreducible essence], and crystallizes it anew in a nobler form” (Burckhardt, 1997, p. 123). Importantly, the initiate requires help “by means of a natural vibration of the soul which awakes….and links the human and cosmic domains” (Burckhardt, 1997, p. 123). The initiate exits this phase when the new structure (i.e., ego, mind, or body) has been placed into order, constructed in its new configuration but not yet fully developed.

The interplay of these three forces in the processes of transformation illustrates the significance of the Divine. If left unaccompanied, humans and nature cannot alone make a hero or a superhero; “True man—the spiritual man—is not given, is *not the result of a natural process*. He is ‘made’ by the old masters, in accordance with the models revealed by Divine Beings and preserved in the myths” (Eliade, 1958/2005, p. 132). With this understanding of how transformation unfolds and how the forces at play commingle to cause evolutionary change, I now turn to the final discussion of how initiates are made into heroes, and how heroes transition to become superheroes.

## Triple transformation: Evolving from Human to Transhuman

In this final section, I complete the framework by describing how an ordinary person becomes a transhuman, meaning living beyond or past the ordinary human experience, a beyond-human, or a superhero, through three progressive, *substantial* transformations. According to spiritual texts (Sri Aurobindo, 1963/1990), psychology (Jung, 1988), Hermetic science (Eberly, 2004), and others, the ultimate purpose of human life is to receive and integrate the Divine into one's body, mind, and spirit, and in so doing, to accomplish the “spiritualization of matter and the materialization of the Holy Spirit” (Eberly, 2004, p. 4). Aurobindo conceptualized a biopsychospiritual pathway of human development he names the *triple transformation* that yields a self-realized and evolved human. In the context of heroism science, the culminating human is a superhero—one with capacities that are both human and beyond human.

One way this goal is conceptualized is through a progression of three great transformations, unfolding in this order: ego, mind, and body (Sri Aurobindo, 1963/1990). The first transformation involves the dissolution or death of the ego because “the hero of yesterday becomes the tyrant of tomorrow, unless he crucifies himself today (Campbell, 1968, p. 353) so the ego can be replaced by the soul, also called the true ego (Sri Aurobindo, 1963/1990). According to this approach to development, the *soul* is the part of the human that is luminous, immortal, and evolves and is personally unique. Mythology reveals that the soul is “identical in form with the universe” (Campbell, 1968, p. 385). The spark of the Divine, seeded in the soul, gives humans a direct connection to the Divine, which is consciousness itself. Individuals can directly sense the soul during moments of inward surrender and unveiled intimate honesty that cause the feeling, ‘this is me.ș The spark of the Divine found at the center of the soul is what “makes [a hu]man an exceptional being” (Sri Aurobindo, 2002, p. 94).

Although it is not within the scope of this paper to attempt to outline processes of ego development, a few key actions relate to this discussion. As a first condition of ego development, the heart, mind, and will/action must grow. Experiences that involve learning about one's self or healing are extremely important, as well as indwelling and self-analysis, because these activities increase self-awareness; “the ego has an imperative need to know it exists” (Temple-Thurston and Laughlin, 2000, p. 57). This growth propels the individual “in a line of an ascent” and can be cultivated by seeking that which is naturally alluring: truth, good, and beauty (Sri Aurobindo, 1963/1990, p. 66). These three turn the individual toward that which is inherently spiritual. These are activities help the ego to see itself more fully and as such aid in its dissolution. For the innermost change, which constitutes the ego's consummation, the ego must make direct contact with “spiritual Reality” because “nothing else can so deeply touch the foundations of our being and stir it or cast the nature by it stir into a ferment of transmutation” (Sri Aurobindo, 1963/1990, p. 66). This transformation constitutes a shift from a self that is founded on ignorance and narrowness, to a self-grounded in conscious awareness of self.

Once the initiate completes their first journey through the entire Figure 8 and the soul has taken on a bodily existence or structure by becoming the central organizing structure of the self; the soul is said to be “firm…fixed and eternal and cannot be burned” (Jung, 1935/1968, p. 27), meaning that the soul cannot be dissolved through the fires of transformation. After this momentous achievement, the individual enjoys “an influx of spiritual experiences of all kinds” (Sri Aurobindo, 1963/1990, p. 71), and all subsequent transformations are much less laborious (Sri Aurobindo, 1972).

The second transformation is of the mind, achieved when “consciousness of the mental creature is…turned wholly into the consciousness of the spiritual being” (Sri Aurobindo, 1963/1990, p. 76) when she is “left alone” with “ultimate openness….into which the mind must plunge alone and be dissolved” (Campbell, 1968, p. 258). The result of this transition endows the mind with “new forces of thought or sight and a greater power of direct spiritual realization that is more than thought or sight, a greater becoming in the spiritual substance of our present being” (p. 75). When the initiate opens up to the “boundless self” (Sri Aurobindo, 1963/1990, p. 73) and when a Divine intervention transfigures ignorance, darkness is dispelled. This transformation is equivalent to what is generally termed ‘enlightenmentș and the capacity to put “all in order” (p. 75).

Only upon the third transformation of the body (having already transformed the ego and mind), does the burgeoning hero-in-transformation become supernatural; through “initiation—dismemberment …resurrection … and new birth, obtaining a new, supernatural body” (Eliade, 1958/2005, p. 108). This perfected state transpires upon these achievements:

To embrace individuality after transcending it is the last and divine sacrifice. The perfect [result] is he who is able to live simultaneously in all these three apparent states of existence, elevate the lower into the higher, receive the higher into the lower, so that he may represent perfectly in the symbols of the world that with which he is identified in all parts of his being, —the triple and triune Brahman (Sri Aurobindo, 1972, p. 396).

This final transformation grounds the spirit (the truest self) into the physical realm of matter by purifying, incarnating, and spiritualizing the body, causing the initiate to become a supramental being—a superman or superwoman. In this great descent, the initiate must “empty” herself “by actively embracing God's absence (kenosis)… [to cause] autonomy of the self in the face of an otherwise all-encompassing other [the Divine] …to free herself from a dependency on ecstatic communion” (Mechthild, 1963/1993, p. 42). This is made possible by “realistic and courageous acceptance of pain and loneliness [due to the absence of an] ecstatic state of fusion with the divine.” (p. 43). The hero-in-transformation strengthens the descent of spirit during her transformation through the continuous “choice to embrace the whole gamut of spiritual experience rather than to depend only on the heights of ecstasy” (p. 43).

When she has ascended to the highest that can happen to her while she is still connected to her body, and sunk down into the greatest depth that she can find, then she is fully grown in virtues and holiness. Then she must be adored with the pain of long waiting (Mechthild, 1963/1993, p. 4).

Aurobindo explains that this third transformation causes one to be *supramental*, to have consciousness of the infinite (Divine), which is not accessible to the ordinary mind that founds itself on consciousness of the finite and uses division and construction as a means of understanding unity. Although the mind is also transfigured in this final transformation, the body is the primary framework that becomes spiritualized with consciousness. The goal of this final transformation is a “soul [that bases its] consciousness, its life, its power and form [body] of manifestation on a complete and completely effective self-knowledge” (Sri Aurobindo, 1963/1990, p. 76), which is attained through continued inward focus and analysis. When an initiate triumphs in her journey of the third transformation, “a new world appears that is born and *contained* in this world of Matter and yet *surpasses* it in its true dynamic nature” (italics added; Sri Aurobindo, 1972, p. 17). Perhaps this is what the contemporary spiritual author and teacher Tolle (2008) intended when he titled is book, “The New Earth” as a causality of human transformation.

## The result: Superheroes as Transhuman

With the final transformation, the hero is made supernatural. The result is “a permanent ascension of the lower ….and permanent descent of the higher into lower” (Sri Aurobindo, 1963/1990, p.74), In other words, the spirit, the true self, descends into and becomes one with the body, and the body ascends and becomes one with the true self. This is effectively a union of soul (the immortal psyche) and spirit (the true self that is already perfect and does not evolve) in the body. The consciousness that emerges from this amalgamation is unbounded, grounded, peaceful, loving, and awake. Jung explains that the superhero is “the circle of the beginning [the origins of the universe] but this circle has now the anima mundi, the soul of the world, which was hidden in the chaos….this time [the circle] is the spiritual body….the redeemed microcosmos” (Jung, 1988, p. 1401). One who achieves this is “capable of resisting all outside influences” (Sri Aurobindo, 2002, p. 108), because she has incarnated. In alchemical and psychological terms, she has become refined, cleared of all “impurities” (i.e., the shadow), leaving only that which is essentially true, uncorrupted, and real—in Hermetic Science terms, that which is irreducible—the enlightened one or the philosopher's stone.

So important is this observation about human transformation that renown anthropologist Mircea Eliade made a brief but emphatic plea about how, after passing through the gauntlet of transformation's dismemberment, the initiate becomes a forerunner of evolution itself:

It must never be forgotten that initiatory death simultaneously signifies the end of the “natural,” noncultural man, and passage to a new modality of existence—that of a being “born to spirit,” that is, a being that does not live solely in an immediate reality. Thus initiatory death forms an integral part of the mystical process by which the novice becomes another, fashioned in accordance with the model revealed by the Gods or the mythical Ancestors. This is as much as to say that one becomes truly a man in proportion as one ceases to be a natural man and resembles a Supernatural Being (Eliade, 1958/2005, p. 132).

In this passionate passage, Eliade illuminates what has been explored here, namely the end of the “natural” human and the birth of a humanity of transhumans—superheroes—through progressive biopsychospiritual processes that repeat three times and results in a spiritualized human. With each transformation, consciousness emerges, transfigures and spiritualizes the ego, mind, and body (i.e., consciousness raising), while conversely, consciousness descends into the reorganizing ego, mind, or body to literally elevate the structure into higher realms while still constituting matter.

Even though spirit or consciousness is always within the individual prior to transformation, after the third transformation, the spirit is actualized, made tangible or real, rendering the superhero in full possession of herself. The super-human result of these three transformations has been recognized by many scholars and thinkers, each with their own designation. Eliade says, “Initiation….reveals a world open to the *transhuman*, a world that, in our philosophical terminology, we should call transcendental” (emphasis added; 1958/2005, p. 132). Campbell says, “God assumes the life of man and man releases the God within himself at the…same sun door though which God descends and Man ascends—each as each other's food (Campbell, 1968, p. 260). He calls the hero transformed a *redeemer* (Campbell, 1968, p. 349), *saint* (p. 354), *king within* (p. 365) or “universal god-man” (p. 389). Hindu tradition names one who has achieved this state is called *Arhat*: one “who is able to live simultaneously in all these three apparent states of existence, elevate the lower into the higher, receive the higher into the lower, so that he may represent perfectly….in all parts of his being—the triple and triune” (Sri Aurobindo, 1972, p. 396); oneness, differentiated oneness, and duality (p. 59). Philosopher Friedrich Nietzsche named his teacher *Superman*, a being who Jung states is, in hermetic terms, “a new unit called the *rotundum*, the roundness, or the round complete thing” (Jung, 1988, p. 1401) that is “the being that can be created by man's making a heroic endeavor to create something beyond himself” (Jung, 1988, p. 49). Hermetic science refers to the one who is self-realized as a master, *magisteria* (Leicester and Klickstein, 1952, p. 22), or *philosopher's stone* (Nietzsche, 1961). Although the stone is one entity, it is “called *Rebis* (two-thing), being composed of…a body and spirit by which the body is dissolved into a spirit” (emphasis added; Henry, 1893/2012, p. 12). Regardless of the name assigned to the new version of human—the transhuman, redeemer, saint, god-man, arhat, rotundum, magisteria, philosopher's stone, Rebis, King or Queen, or superhero—it is certain that she is an entirely new human. The transformed individual is one but consists of two: human and superhuman. Said in a different way,

Everything existing phenomenally or, as we shall say, symbolically, two parts, the thing in itself and the symbol, Self and nature, res (thing that is) and factum (thing that is made), immutable being and mutable becoming, that which is supernatural in it and that which is natural. (Sri Aurobindo, 1972, p. 52)

As exhibited in this paper, the two-thing—the human-superhuman—lives in three worlds, the lower, middle, and upper. When an initiate realizes a complete transformation, an entire Figure-8 for the first time, the process places them in relationship with three realms of the lower, middle earthly, and upper.

## Conclusion

If we are to better apprehend the nature of heroism, I argue, we must go beyond analysis of its processes (the content corresponding to how transformation unfolds), which have been explored by Jung (1988), Turner (1969), Eliade (1958/2005), Ouspensky (1949/2001) and Sri Aurobindo (1963/1990), among others. We must also examine thoroughly the contextual elements, what is natural and above nature, that affect the process. Just as an oceanographer must know meteorology and astronomy to better know the ocean, I uphold that to study heroism means we also scrutinize the forces that act upon the initiate during transformation. Following this line of thought, this paper has been so devised.

Founded on the scholarship of seminal authors of depth psychology (Jung, 1988), Hindu spirituality (Sri Aurobindo, 1972), anthropology (Turner, 1969), physics (Prigogine, 1997), Hermetic science (Eberly, 2004), mythology (Campbell, 1968), and other disciplines, this paper demystifies how an ordinary person becomes a hero by deconstructing the characteristics of transformation as a key function of human psychospiritual evolution. The primary proposition is that human transformation unfolds through both natural and supernatural phenomena and produces a transhuman or superhero: one who is natural (bound by nature) and supernatural (beyond nature).

The discourse begins at the level of limitations, by describing four key “laws” that govern the realm of matter and ground the budding hero as an earth-bound human, and then defining four supernatural abilities the hero-initiate must cultivate and enact in order to transform. The next section provides an overlay of natural and supernatural processes upon the Figure-8 Model of Transformation (Ross, 2017), to “map” when the initiate can expect to receive the support of natural processes, when to be vigilant for transcendental or Divine intervention, and when to courageously meet challenges with supernatural action. This paper culminates with an explanation of three progressive, substantial initiatory transformation cycles—the triple transformation (Sri Aurobindo, 1963/1990)—that transmute an ordinary person into a hero and the hero into a super, transhuman.

We will recognize her and know him because, “The will of a single [super]hero…can breathe courage into the hearts of a million cowards….The thought of a solitary [superhero] can become, by exercise of selfless and undoubting will, the thought of a nation” (Sri Aurobindo, 1972, pp. 178–179). The world has for example, known a number of individuals who appear to have demonstrated such resplendence; Sojourner Truth, Mahatma Gandhi, Susan Cady Stanton, Nelson Mandela, Corazon Aquino, Genghis Khan, and Joan of Arc, to name a few.

With this crowning achievement, the superhero is made real, and a vision deciphered by Joseph Campbell comprehensible; “the cosmogonic cycle is now to be carried forward, therefore, not by the gods, who have become invisible, but by the heroes, more or less human in character, through whom the world destiny is realized” (1968, p. 315). Hero-making serves a far greater and hallowed purpose than nobly transforming one's own self and motivating beloved others to do the same. When we dare to be guided into action by our cherished heroes, we engage in an important evolutionary compulsion. We need only follow the pathway already laid before us as our ordinary, extraordinary life.

## Author Contributions

The author confirms being the sole contributor of this work and has approved it for publication.

### Conflict of Interest Statement

The author declares that the research was conducted in the absence of any commercial or financial relationships that could be construed as a potential conflict of interest.

## Footnotes

^1^As the assumed default pronoun is “he”, “he or she” is awkward, and gender is a spectrum, in this paper “she” is used to refer to and include individual humans of all genders.
